# Reporting of euthanasia and physician-assisted suicide in the Netherlands: descriptive study

**DOI:** 10.1186/1472-6939-10-18

**Published:** 2009-10-27

**Authors:** Hilde Buiting, Johannes van Delden, Bregje Onwuteaka-Philpsen, Judith Rietjens, Mette Rurup, Donald van Tol, Joseph Gevers, Paul van der Maas, Agnes van der Heide

**Affiliations:** 1 Erasmus MC, University Medical Center Rotterdam, Department of Public Health, Rotterdam, the Netherlands; 2 University Medical Center Utrecht, Julius Center for Health Sciences, Utrecht, the Netherlands; 3 VU University Medical Center, Department of Public and Occupational Health, EMGO+ Institute for Health and Care Research, Amsterdam, the Netherlands; 4 University Medical Center Groningen, Department of Health Sciences, Section Metamedica, Groningen, the Netherlands; 5 Academic Medical Center, Department of Social Medicine, Health Law Section, Amsterdam, the Netherlands

## Abstract

**Background:**

An important principle underlying the Dutch Euthanasia Act is physicians' responsibility to alleviate patients' suffering. The Dutch Act states that euthanasia and physician-assisted suicide are not punishable if the attending physician acts in accordance with criteria of due care. These criteria concern the patient's request, the patient's suffering (unbearable and hopeless), the information provided to the patient, the presence of reasonable alternatives, consultation of another physician and the applied method of ending life. To demonstrate their compliance, the Act requires physicians to report euthanasia to a review committee. We studied which arguments Dutch physicians use to substantiate their adherence to the criteria and which aspects attract review committees' attention.

**Methods:**

We examined 158 files of reported euthanasia and physician-assisted suicide cases that were approved by the review committees. We studied the physicians' reports and the verdicts of the review committees by using a checklist.

**Results:**

Physicians reported that the patient's request had been well-considered because the patient was clear-headed (65%) and/or had repeated the request several times (23%). Unbearable suffering was often substantiated with physical symptoms (62%), function loss (33%), dependency (28%) or deterioration (15%). In 35%, physicians reported that there had been alternatives to relieve patients' suffering which were refused by the majority. The nature of the relationship with the consultant was sometimes unclear: the consultant was reported to have been an unknown colleague (39%), a known colleague (21%), otherwise (25%), or not clearly specified in the report (24%). Review committees relatively often scrutinized the consultation (41%) and the patient's (unbearable) suffering (32%); they had few questions about possible alternatives (1%).

**Conclusion:**

Dutch physicians substantiate their adherence to the criteria in a variable way with an emphasis on physical symptoms. The information they provide is in most cases sufficient to enable adequate review. Review committees' control seems to focus on (unbearable) suffering and on procedural issues.

## Background

At present, physician-assistance in dying is known to be provided in several countries in varying frequencies [1-4]. In the Netherlands, euthanasia is defined as deliberately ending a person's life at the person's request. In physician-assisted suicide, the person self-administers medication that is prescribed by a physician. Euthanasia and physician-assisted suicide are allowed provided that a physician performs the act while adhering to specific requirements. Euthanasia and/or physician-assisted suicide is also legally allowed in Belgium, Luxembourg and the US states of Oregon and Washington [5-8] and discussions about the legalization of physician-assisted dying are going on in other countries, such as the UK, Spain, France, Columbia and Australia [9-13]. Debates about legalization often relate to concerns about whether it is possible to keep the practice of physician-assisted dying within agreed borders [14,15]. Concerns relate to the risk that vulnerable people may be or feel coerced to request assistance in dying, that alternatives to assistance in dying are lost out of sight, or that deciding to provide assistance in dying becomes too 'easy' an option without careful consideration of alternatives.

In the Netherlands, physicians have to report euthanasia and physician-assisted suicide to enable review by one of five regional multidisciplinary review committees. They should comply with criteria of due care that have been developed by the courts during the preceding decades and are generally considered to be a summary of case law [16]. These criteria require a physician to assess that (1) the patient's request is voluntary and well-considered, (2) the patient's suffering is unbearable and hopeless, (3) the patient is informed about his situation and prospects and (4) there are no reasonable alternatives. Further, (5) another, independent physician should be consulted and (6) the termination of life should be performed with due medical care and attention [17]. To demonstrate their compliance with these criteria, physicians have to submit a detailed report, which describes their way of acting and its circumstances. This report is usually based on a standard form that contains both open and closed questions regarding the criteria of due care. Review committees have to assess whether the physician acted in accordance with the criteria. They do so by scrutinizing the physician's report, and, if necessary, by asking the physician to supplement this report either orally or in writing, or by obtaining information from other persons involved. The Belgian Euthanasia Act, that legalizes euthanasia since 2002, is largely similar to the Dutch Euthanasia Act [18]: the Belgian Act includes similar criteria of due care but review is done by one multidisciplinary committee. Luxembourg legislation (2008), draws heavily on the Belgian experience [5].

These Acts differ from the Oregon Death with Dignity Act, that legalizes physician-assisted suicide since 1997 [8]. To request a prescription for lethal medication, the Oregon Act requires that the patient is an adult resident of Oregon who is capable and who has an illness that is expected to lead to death within six months. To obtain the lethal medication the patient should make one written and two oral requests (separated by at least 15 days) to his or her physician. The patient's primary physician and a consultant are required to confirm the diagnosis of a terminal condition and the prognosis, determine that the patient is capable, and refer the patient to a psychiatrist or clinical psychologist for further evaluation, if either believes that the patient's judgment is impaired by depression or other psychiatric/psychological disorder. The primary physician should also inform the patient of all feasible alternatives [19]. If the patient meets the eligibility criteria and the physician writes a prescription, physicians have to report to the Oregon Health Division which lethal medications were prescribed. They further have to indicate that they fulfilled the requirements by checking the boxes in the attending physician's compliance form [20]. After receiving the report of the death of the patient, the Health Division asks the reporting physician whether the patient indeed had died from the medication. The law and requirements for the Washington Death with Dignity Act are virtually identical to the Oregon Act [21].

The purpose of reporting and reviewing practices of euthanasia and physician-assisted suicide is to evaluate how the norms laid out in the laws and regulations are being handled in actual practice. External review enables countries to evaluate whether current regulation suffices to restrict euthanasia to cases that meet the criteria, to see where potential problems occur and to educate physicians to comply with the rules. Various studies have been performed about how physicians perceive the patient's suffering and in what situations patient's requests result in euthanasia [22-24]. In the Netherlands as well as Oregon, physicians have been shown to be motivated to engage in euthanasia because of their patients' disease-related experiences, such as severe pain, functional loss, discomfort, fatigue, and expressed loss of dignity of the patient.

The ethical foundation of the five Acts described, is a combination of respect for autonomy and obligations of beneficence. However, for the Dutch, Belgian and Luxembourg Acts, addressing the patient's suffering is the most important principle underlying the Act. The Oregon and Washington Acts, on the other hand, put emphasis on patients' rights and on helping patients to maintain control and independence. Whether or not these differences in emphasis lead to differences in practice and in the review procedure is unclear.

For Dutch review committees, physicians' reports are an important basis for their assessment whether the criteria of due care have been met or not. However, the content of what physicians report about the criteria of due care and how review committees judge this information is for the most part unknown. We studied which arguments Dutch physicians use to substantiate that they have adhered to the requirements of due care and which aspects attract review committees' attention. Furthermore, we compared our findings with existing information about other external review procedures and reflect on whether a different procedure would result in a different focus of attention.

## Methods

### File content

The files of euthanasia and physician-assisted suicide are archived at the offices of the 5 regional euthanasia review committees. The files comprise the physician's report, one or more reports of the consulted physician and the verdict of the review committees. The file may also include copies of medical files and letters from clinical specialists. The physician's report nearly always consists of a 'standard form' that contains various questions about the criteria of due care. This form is developed by the review committees and the Dutch Ministry of Health, Welfare and Sports. It is available via the Royal Dutch Medical Association and the Ministry. In 2002 the initial standard form had been replaced by a newer version that contained more specific questions about the 'unbearableness' and the 'hopelessness' of the patient's suffering, and about how the patient was informed about his situation. Further, the questions whether the patient's request was 'voluntary' and whether there were possibilities to relieve the patient's suffering were somewhat rephrased as well.

### Study design

In 2005, 1933 euthanasia cases were reported to a review committee [25]. As part of a larger study aimed at evaluating the Dutch Euthanasia Act, we studied the physician's reports and the verdicts of the review committees in 243 files of euthanasia or physician-assisted suicide cases that were performed in 2005 [26]. The sample consisted of two parts. The first part included all 115 cases in 2005 (6% of all cases) where review committees had had doubts or questions and asked the reporting physician to provide additional information. The second part of our sample included 'the last' 117 reported cases in 2005, which we presumed to be representative for all reported cases. This part of the sample was stratified for the five review committees. Strict anonymity of the patient and the physician was guaranteed.

In this study, the sample was restricted to 158 cases where reporting physicians had used the latest version of the standard form, because this version will also be the basis for future reports on euthanasia or physician-assisted suicide. We studied 75 cases where review committees had had doubts or questions and 83 cases that belonged to 'the last' reported cases in 2005. Weighting of the results to correct for differences in sampling fractions did not appear to affect the results; we therefore only report unweighted results. All studied cases were approved by a review committee. According to Dutch policy, the study did not require review by an ethics committee because the data collection was anonymous with regard to the deceased patient and the attending physician.

### Checklist

To study the files, we developed a checklist that covered all topics in the standard form. In this paper we only use data related to the six criteria of due care. The specific questions are either included in the Tables or separately mentioned in the results section. The checklist was piloted by 2 researchers who scored 10 files each. The outcome of this pilot necessitated a few changes in the wording of the final version of the checklist. Eight researchers were involved in scoring the files. During the scoring of the files, issues that were unclear were always discussed and communicated with all researchers. Fourteen files were double checked to estimate the interrater reliability. The average agreement for 20 randomly chosen variables was 91% (minimum agreement 75% vs. maximum agreement 100%).

## Results

### The patient's request

In all studied cases except one, physicians reported that the euthanasia request had been made by a patient who was fully aware of his physical situation (Table 1). Physicians often substantiated this conclusion by stating that the patient had been clear headed while expressing his or her request (65%) and/or had repeated the request several times (23%). In all cases, physicians reported that the euthanasia request had been made without pressure from others. In 97%, a written euthanasia declaration, that is not obligatory under the Dutch legislation, was available; in one case the physician reported that the patient had not been capable to sign the declaration anymore, in one case information about whether or not there had been a written euthanasia declaration was missing in the physician's report, in one case it concerned a declaration about general medical care instead of euthanasia and in one case it was not clearly specified in the report why the euthanasia declaration was missing.

**Table 1:** Characteristics of the patient's request.(Questions phrased from the standard form of the reporting physician)

	n = 158 %
*Was the patient, while expressing the request, fully aware of the implications of his or her request and of his or her physical situation?*	
	
No	1^1^
Yes	99
	
*If yes, from what circumstances did you make these conclusions? *^2^	
	
Patient was clear headed	65
Patient's request was repeated several times	23
Patient had no mental problems	13
Patient was aware about his situation and prospects	10
Physician knew the patient very well	4
Other^3^	15
Not clearly specified in physicians' report	5

*Are there indications that the patient's request was expressed under pressure from others?*	
	
No	100

*Was a written euthanasia declaration available?*	
	
Yes	97

1 case.

2. More than one answer could be given, open question.

3. Other includes: Family was convinced that the request was well-considered, patient's request had been judged by another physician, availability of an advance directive, patient always wanted to decide for himself.

### The patient's suffering

Table 2 presents the arguments given why the patient's suffering was considered to be 'unbearable' or 'hopeless'. In 62%, physicians reported that the patient's suffering was 'unbearable' because there were one or more physical symptoms; they most frequently mentioned pain (32%), dyspnea (22%), fatigue (15%) or nausea (15%). A third of all physicians reported that function loss had contributed to unbearable suffering, such as being bedridden (19%) or having a decreased appetite or capacity to eat or swallow (10%). In 63% physicians mentioned 'other aspects'; these included increased dependency (28%), deterioration (15%) and more rare aspects (16%), such as loneliness, being a burden to relatives and being mentally exhausted. Physicians most often based the 'hopelessness' of the suffering upon the "absence of treatment alternatives" (32%), "absence of curative treatment alternatives" (28%), or "absence of treatment alternatives to relieve the patient's symptoms", or combinations of these (14%).

**Table 2:** Arguments for patient's suffering being unbearable or hopeless. (Questions phrased from the standard form of the reporting physician)

	**n = 158**^**1**^ %
*Could the suffering be considered unbearable? Please motivate*.	
	
**Symptoms**^**2**^	**62**
Pain	32
Dyspnoea	22
Fatigue	15
Nausea/vomiting	15
Incontinence/diarrhoea/constipation	6
Cachexia	6
Confusion	3
Fear	3
Other^3^	9
**Function loss**^**2**^	**33**
Bedridden	19
Appetite/thirst/eating- and swallowing capacity	10
Language	4
Other^4^	4
**Other aspects**^**2**^	**63**
Dependency	28
Deterioration/general malaise	15
Hopelessness, no treatment possible	13
Loss of autonomy/identity	4
Loss of dignity	2
Mentally exhausted	7
Other^5^	16

*Could the suffering be considered hopeless? Please motivate*.	
	
No treatments possible	32
No curative treatments possible	28
No treatments to relieve symptoms possible	3
No curative treatments + treatments to relieve symptoms possible	11
Short life expectancy	8
Other^6^	9
Not clearly specified in the report	8

1. In 8 cases (4%) the nature of patient's suffering was explained, but no explicit arguments for the suffering being unbearable were given.

2. More than one aspect could be mentioned.

3. Other include: decubitus, edema, epileptic insults, itch, and cough.

4. Other include: cognitive function, sleeping problems and general physical functioning.

5. Other include: loneliness, to be a burden to relatives, losing interest, mental suffering, no quality of life.

6. Other include: no differentiation between unbearable and hopeless suffering, worsening expected.

### The information provided to patients about their situation and prognosis

In the standard form, one question pertains to the information provided to the patient: *'How was the patient informed about his prognosis (current situation, course, prognosis, etc)?' *Physicians mentioned in 77% of cases that they themselves had informed the patient (not in table). In 58%, they reported that other physicians (mainly medical specialists) had informed the patient. In 14%, they used other terms to describe how the patient was informed such as "Extensively discussed orally", and "completely". In a few cases (3%), it was reported that patients had gathered their information through written material on the internet (always in combination with information from reporting physicians).

### Medical treatment/care

Table 3 shows that physicians in almost all cases reported to have applied palliative care options, most frequently medication (89%) and sometimes radio- or chemotherapy (21%). 'Other' palliative care options (46%) often concerned administering oxygen, nutrition or hydration, or artificial respiration. In 35% of all cases it was reported that there had been options to relieve the patient's suffering that were not applied. These most often involved the administration of sedatives (10%) or pain medication (11%). In 81% of the cases where these alternatives had been present, physicians reported that the patients had refused them.

**Table 3:** Characteristics of the presence of reasonable alternatives. (questions phrased from the standard form of the reporting physician)

	n = 158 %
*What had been done in terms of palliative care?^1^*	
	
Medication	89
Radio- or chemotherapy	21
Other^2^	46
Not clearly specified in the report	1
	
*Were there (other) possibilities to relieve the patient's suffering?^1^*	
	
Yes:	35^3^
Administration of sedatives	10
Other pain medication	11
Radio- or chemotherapy	3
Intensive home care/family care	2
Other	10
	
*How did the patient feel about these alternatives?*	
	
	n = 56
Positive	4
Negative	81
Other	13
Not clearly specified in the report	2

1. One or more answers could be given.

2. Other include: oxygen administration, artificial respiration, artificial administration of food and fluids, blood transfusions, home care, surgery, stoma, administration of sedatives, talks with the patient.

3. In three cases, the question was answered affirmatively but not further explained.

### The consultation

Physicians consulted more than one physician in 29% of all cases (Table 4). The consultant had been a 'SCEN-physician' (Support and Consultation for Euthanasia in the Netherlands) in 85% of all cases. The nature of the relationship between the reporting physician and the consultant was not always clearly specified. In 39%, physicians indicated that there was no earlier relationship between the reporting physician and the consultant. Physicians indicated they had known the consultant beforehand in 21% of the cases. In 9% it was unclear whether the physician knew the consultant beforehand or not (i.e. "a colleague" or "professional relationship") and in 6% they reported that it had been a SCEN-physician without further specifying the relationship. If only one consultant had been consulted, these percentages were comparable except that the consultant less often had been a known colleague (data not in Table).

**Table 4:** Characteristics of the consultation. (Questions phrased from the standard form of the reporting physician)

	n = 158 %
Number of physicians that had been consulted	
One	71
Two	22
Three	7
	
*Which physicians were consulted? In the capacity of:*	
	
SCEN-physician^2^	85
General practitioner	18
Medical specialist	30
Other	3
	
*Was/were they already involved in the care for the patient?*	
	
Yes	1
One involved, the other not	18
No	80
Not clearly specified in the report	2
	
*What was the nature of the relationship towards the reporting physician?*	
	
Unknown colleague	39
Unclear whether colleague is unknown or not	9
Known colleague ^3^	21
'SCEN-physician'	6
Other	10
Not clearly specified in the report	24

1. More than one physician could have been consulted.

2. SCEN = Support and Consultation for Euthanasia in the Netherlands. A 'SCEN-physician' is a physician who has received formal training in consultation and participates in a formal network of consultants.

3. Colleague own practice/partnership/other collaboration (8%). Familiar colleague not related to own practice/partnership/other collaboration (13%).

### The performance of euthanasia

We used one question in the standard form that related to the performance of euthanasia: '*Which medication was used and how was life ended?' *In 76% of all cases, physicians reported to have administered a barbiturate followed by a muscle relaxant (not in table). In 19%, they reported to have administered a barbiturate only; these concerned all physician-assisted suicide cases and some cases of euthanasia. In 3% they only reported a muscular relaxant and in 3% physicians did not specify which medication they had used.

### Questions for additional information from the review committees

Table 5 shows that if review committees asked for additional information, they most frequently asked for additional information about the consultation (41%), especially with regard to the independency of the consultant (19%) and the quality of the consultant's report (12%). Questions about why the patient's suffering was considered 'unbearable' or 'hopeless' were also frequently asked (32%). Furthermore, review committees relatively often asked about the type of medication (13%) and about topics not directly related to the criteria of due care, such as the quality of the physician's report (13%) and about other aspects (11%) such as whether the reporting physician was the same physician who had performed euthanasia and the reasons for the high number of independent consultations for one patient.

**Table 5:** Topics about which review committees asked for additional information (As described in the verdicts of the review committees) n = 75.

	%	(n)	%
**The patient's request**^**1**^	11	(8)	
▪ Being well-considered			8
▪ Voluntariness			9
**The patient's suffering**^**1**^	32	(24)	
▪ Further specification of (unbearable) suffering			23
▪ Course of disease			12
▪ Patient was (sub) comatose			4
▪ Other			4
**Informing the patient about their situation and prognosis**		-	
**The presence of reasonable alternatives**	1	(1)	
**The consultation**^**1,2**^	41	(31)	
▪ Quality of consultant's report			12
▪ Independency of consultant			19
▪ Moment of consultation			9
▪ Quality of consultation			1
▪ Other			4
**Performance of euthanasia and physician-assisted suicide**^**1**^	17	(13)	
▪ Type of medication			13
▪ Physician's attendance			3
▪ Other			3

**Other topics**^**1**^	21	(16)	
▪ Decision-making of the physician			1
▪ Quality of physician's report			13
▪ Other			11

1. More than one answer could be given.

2. In 70% of the cases, the reporting physician was also involved in the question for additional information from the review committees.

## Discussion

This study demonstrates that physicians report about cases in a variable way for most of the criteria for due care. Some criteria are hardly substantiated (voluntariness of the request, patient being well informed) and others are substantiated mainly by mentioning physical aspects (unbearable suffering). This study demonstrates that *if *review committees asked the reporting physician to provide further information, it primarily concerned the 'unbearableness' of the patient's suffering and the consultation of an independent physician.

### The patient's request

In virtually all cases, physicians reported that the request had been voluntary and well-considered. A previous study showed that not granting a request for euthanasia or physician-assisted suicide is frequently due to the request not being well-considered according to the physician [22]. A voluntary and well-considered request is considered a condition sine qua non for euthanasia. However, physicians did not uniformly argue *why *they thought the request was well-considered and review committees rarely asked for additional information about this requirement. Possibly, the information was convincing enough for review committees to guarantee that the patient's request was voluntary and well-considered if the file did not contain information suggesting otherwise. This finding is consistent with an interview study among review committee members that showed that committee members virtually never experience problems with judging the patient's request [26]. On the other hand, elsewhere we found that physicians frequently report to experience difficulties in determining whether the request was voluntary and well-considered [27]. Such difficulties, however, do not seem to be present in the reporting discourse, nor in the requests for additional information of the committees.

### The patient's suffering

Various arguments for the patient's suffering being 'unbearable' were given. Physicians often mentioned physical symptoms like pain, but function loss and other aspects related to the patient's suffering were mentioned as well. As reported previously, physicians who receive requests for euthanasia or physician-assisted suicide frequently experience problems with assessing the 'unbearableness' of the patient's suffering [27]. Whether or not a patient's situation is unbearable is to a large extent a matter of the patient's subjective experience and perspective, which can comprise more than physical symptoms alone [28]. A questionnaire study among physicians showed that pain had been among the reasons to perform euthanasia in 47% of cases nearly always in combination with other reasons. Other reasons, such as the lack of prospects of improvement (85%) or the patient's loss of dignity (60%) were more common than pain [26]. In addition, patients' *requests *for euthanasia are also often reported to be grounded in fear of losing dignity or autonomy and to a lesser extent by pain [22,29]. In their report to review committees however, physicians seldom reported loss of dignity but they relatively often mentioned physical aspects, such as pain and dyspnea. Probably, physicians attach much value to physical symptoms in their report because these can be more easily and objectively judged within their own medical-professional domain. Furthermore, based on a court decision of the Dutch Supreme Court (2002) that stated that in case of euthanasia the suffering should predominantly result from a medically classifiable disease or disorder [30], some physicians may assume that review committees consider physical symptoms as an important prerequisite for euthanasia. However, review committees relatively often asked for additional information about the 'unbearableness' of the patient's suffering. This suggests that besides physical symptoms other factors are important for their judgment.

### The information provided to patients about their situation and prognosis

In general, physicians briefly addressed the requirement that the patient should be well-informed about his situation and prospects. In the cases studied, the review committees never asked for additional information about this requirement. The general Dutch law on patient-physician relationships, the Act on the Medical treatment agreement (In Dutch: WGBO), that states that every patient should be well-informed about his situation and prospects before deciding about treatment, is widely known and included in many checklists and guides by the Royal Dutch Medical Association [31]. Possibly, both physicians and review committees assume that informing the patient is a part of normal medical practice that has to be elaborated on in the review process only in case of clear indications of problems.

### The absence of 'reasonable' alternatives

In 35% of all cases, physicians reported that at the time of the decision-making about euthanasia there had been (other) possibilities to relieve the patient's suffering that had not been applied. The majority of patients had refused these alternatives. The Euthanasia Act states that there should be no 'reasonable' alternative available to relieve the patient's suffering. However, the question in the standard form with regard to the presence of possibilities to relieve the patient's suffering does not include the adjective 'reasonable'. Physicians may thus not fully address this criterion in their reports as it is formulated in the Euthanasia Act. According to parliamentary proceedings, a patient may refuse treatment or palliative care; however, if the intervention proposed is not very invasive the physician may conclude that there is a reasonable alternative and that euthanasia or assistance in suicide is therefore not justified. However, our data show that a negative attitude of the patient plays an important role in deciding whether or not an alternative is 'reasonable', for both physicians and review committees. An interview study with committee members [26] showed that committees frequently have discussions about this requirement but often choose to go along with the patient's refusal. In their annual accounts, review committees virtually always address this topic; they stress the importance of obtaining information from the reporting physician about *why *they thought the proposed alternatives were considered to be unreasonable for a particular patient. Nevertheless, despite the fact that the Dutch euthanasia policy is based on the principle that euthanasia and physician-assisted suicide are only acceptable when the patient's suffering cannot otherwise be relieved, review committees virtually never ask the reporting physician to substantiate the lack of alternatives.

### The consultation

Whether or not physicians consulted a totally independent second physician was not made fully clear in every report. In more than 80% of all cases, physicians reported that the consultant was a SCEN-physician and 6% used the word 'SCEN-physician' to describe their relationship with the consultant. SCEN-physicians have received training on medical, ethical and legal aspects of euthanasia and end-of-life care and participate in a formal network to provide independent and high-quality consultations in cases of requests for euthanasia or physician-assisted suicide. Implementation of SCEN-consultations contribute to the quality of the consultation for euthanasia or physician-assisted suicide as has been shown in another study [32] partly because it stands for the independence of the consultant. The fact that the independent consultation most frequently received review committees' attention either suggests insufficient substantiation of this requirement or relatively high importance that review committees attach to this specific requirement.

### Performance of euthanasia or physician-assisted suicide

In general, physicians reported to act according to the guideline of the Royal Dutch Association for the Advancement of Pharmacy that recommends the use of a barbiturate to induce a coma, followed by a muscle relaxant to induce the patient's death [33]. It seems likely that the physicians' report about *euthanasia *was incomplete in cases where only muscle relaxants were mentioned. The requirement that the termination of life should be performed with due medical care and attention especially led to additional questions from the review committees with regard to the type of medication, the use of muscle relaxants without sedatives for instance, may be very distressing for the patient.

Some limitations need to be taken into account. First, we analyzed the files with a checklist. Although we piloted the checklist and discussed possible interpretation problems during the data collection, we cannot preclude that the investigators' interpretation of certain information has influenced the results. Second, we did not investigate the consultant's report or other information in the files of reported cases and this study therefore does not provide a complete picture of the review process. Our main aim was to provide insight in the discourse between reporting physicians and review committees, not to review the reporting system as a whole. This study provides information about what physicians *report *about cases of euthanasia or physician-assisted suicide. Therefore, this study should not be seen as a description of practice but as a description of how physicians describe and interpret their acts.

## Conclusion

The reported cases in 2005 represent a substantial amount (80%) of all euthanasia cases in the Netherlands in that year; studying the files therefore gives important insight in the practice of euthanasia [34]. Physicians substantiate the information provided to the patient and the performance of euthanasia in a rather straightforward and uniform way, but their substantiation is more variable for the patient's request, the patient's suffering, the absence of reasonable alternatives and the consultation. The variation we found is firstly due to variation in clinical situations, in particular with regard to the patient's suffering and the absence of reasonable alternatives. However, what physicians report may also be influenced by differences in knowledge of and viewpoints on euthanasia and the Euthanasia Act, and by uncertainty about how to deal with these criteria. It should be stressed that problems with the interpretation of some criteria of due care are not necessarily a negative finding. In the Act, criteria related to the patient's request, the patient's suffering, and the absence of reasonable alternatives are purposefully framed in open general terms. As such, the Act allows physicians and review committees to newly interpret the criteria in every new case, taking into account the specific circumstances of that case. Furthermore, the questions in the standard form concern both specific closed questions that call for straightforward answers, and open questions that often result in more variable answers. The standard form may thus also influence physicians' reports on the criteria of due care. It seems that there is room for improvement of this form, especially for questions concerning the reasonableness of the alternatives, and the independence of the consultation. For treatment alternatives for instance, the question should be formulated more clearly to be able to assess whether these alternatives were considered 'reasonable' by the physician him- or herself. Recently, (June 2009), a new report form has become available [35]; the impact of these changes (which are partly in accordance with our recommendations) need to be awaited and studied.

For review committees, the standard form that is filled out by the reporting physician generally gives sufficient information to form their judgment; review committees asked for additional information in only 6% of all reported cases. We found elsewhere that review committees basically trust the reporting physicians [26], which may indicate that those 6% involve cases with clear inconsistencies or missing information. Possibly, the committees assume that reporting a case already reflects physicians' intention to act according to the legal criteria. A certain level of trust between review committees and reporting physicians is a prerequisite for an adequate reporting procedure, as this would stimulate physicians to report their acts. Review committees seem to mainly verify that the physician acted with due care, rather than trying to falsify this by looking for incongruent information. They concentrate their additional inquiries on two specific criteria; a subjective one (the patient's suffering) and a procedural one (the consultation), but hardly ask questions about the physical condition of the patient and the presence of possible alternatives. Possibly, their basic attitude of trust in the reporting physician is primarily related to criteria that physicians can assess within their own medical professional domain. Unbearable suffering is the most debated requirement, being subjectively and openly framed. Review committees possibly view their role as more relevant for this specific criterion than for criteria that mainly ask for profound medical knowledge.

Our results show that the Dutch review procedure seems to concentrate on the criterion of (unbearable) suffering and on procedural issues. US legislations do not contain criteria concerning the patients' degree of suffering: the patient's medical situation is addressed in the criterion concerning the patient's life expectancy which should be six months or less. In actual medical practice, the characteristics of patients who died as a result of euthanasia are rather similar in the Netherlands and Oregon and in both countries reported cases are rarely not approved. Differences in the formulation of due care criteria concerning the patient's medical situation apparently only have a limited impact in the practice of physician-assisted dying.

## Competing interests

The authors declare that they have no competing interests.

## Authors' contributions

AvdH, JvD, BOP, MR, DvT, JR, JG, PvdM and HB participated in the design of the study and the acquisition of data. AvdH, HB and JR performed the statistical analysis. AvdH, JvD, BOP, MR, DvT, JG, PvdM, JR and HB interpreted the data. AvdH, JvD and HB were involved in writing of the paper. All co-authors read the paper for important intellectual content and gave their approval of the manuscript.

## Funding/support

This study was supported by a grant from ZonMW, the Netherlands Organization for Health Research and Development.

## Pre-publication history

The pre-publication history for this paper can be accessed here:

http://www.biomedcentral.com/1472-6939/10/18/prepub
